# Directed Evolution of Recombinant C-Terminal Truncated *Staphylococcus epidermidis* Lipase AT2 for the Enhancement of Thermostability

**DOI:** 10.3390/ijms18112202

**Published:** 2017-11-04

**Authors:** Jiivittha Veno, Nor Hafizah Ahmad Kamarudin, Mohd Shukuri Mohamad Ali, Malihe Masomian, Raja Noor Zaliha Raja Abd. Rahman

**Affiliations:** 1 Enzyme and Microbial Technology Research Centre, Universiti Putra Malaysia, Serdang 43400, Selangor, Malaysia; jiivi87@yahoo.com.sg (J.V.); hafizah_kamar@upm.edu.my (N.H.A.K.); mshukuri@upm.edu.my (M.S.M.A.); masomian2000@yahoo.com (M.M.); 2 Department of Microbiology, Faculty of Biotechnology and Biomolecular Science, Universiti Putra Malaysia, Serdang 43400, Selangor, Malaysia; 3 Department of Biochemistry, Faculty of Biotechnology and Biomolecular Science, Universiti Putra Malaysia, Serdang 43400, Selangor, Malaysia

**Keywords:** directed evolution, Staphylococcal lipases, thermostability, characterization, circular dichroism

## Abstract

In the industrial processes, lipases are expected to operate at temperatures above 45 °C and could retain activity in organic solvents. Hence, a C-terminal truncated lipase from *Staphylococcus epidermis* AT2 (rT-M386) was engineered by directed evolution. A mutant with glycine-to-cysteine substitution (G210C) demonstrated a remarkable improvement of thermostability, whereby the mutation enhanced the activity five-fold when compared to the rT-M386 at 50 °C. The rT-M386 and G210C lipases were purified concurrently using GST-affinity chromatography. The biochemical and biophysical properties of both enzymes were investigated. The G210C lipase showed a higher optimum temperature (45 °C) and displayed a more prolonged half-life in the range of 40–60 °C as compared to rT-M386. Both lipases exhibited optimal activity and stability at pH 8. The G210C showed the highest stability in the presence of polar organic solvents at 50 °C compared to the rT-M386. Denatured protein analysis presented a significant change in the molecular ellipticity value above 60 °C, which verified the experimental result on the temperature and thermostability profile of G210C.

## 1. Introduction

Lipases are versatile due to their capability to react in both aqueous and non-aqueous environments. Since many industrial applications operate at temperatures above 45 °C, lipases should preferably have enzymatic activity and thermal stability approximately at 50 °C [1]. The thermostability of an enzyme is closely associated with its expression, folding, activities, and functions, thereby thermal stability is a fundamental property of an enzyme [2]. Thermostable lipases can be exploited in food processing, pharmaceutical industries, biopolymers; organic synthesis, biodiesel production, and the oleochemical and pulp industries [3,4,5].

Lipases with increased thermal properties are needed for industrial lipase-catalyzed reactions as they can increase the conversion rate of lipid substrates, especially with a high melting point, resist chemical modifications at elevated temperature and prevent contamination by microorganism [6,7]. Organic tolerant lipases with increased thermal properties are needed for the industrial lipase-catalyzed reactions as they can resist chemical modifications, prevent contamination by microorganism and also increase the conversion rate of lipid substrates, especially with a high melting point [6,7]. Although, many researchers have characterized thermostable microbial lipases, only a few lipases have been reported to possess both thermostable and organic solvent-tolerant properties.

Up to now, there are no studies describing thermostable lipases from *Staphylococcus* sp. These are rarely exploited in industrial processes because of their relatively low catalytic activities and stability under conditions that are required for industrial applications such as high temperatures, non-aqueous solvents, or extreme pH values [8]. The Staphylococcal lipases can be engineered to alter their properties for better performance in the practical applications in the aspect of quality and quantity [9].

Directed evolution has rapidly emerged as a most favorable method for enhancing the biocatalysis activity of a microbial enzyme to generate variants with desired characteristics [10]. This strategy introduces random mutation(s) along the sequence [11]. Recently, many enzymes were being engineered via directed evolution to have unique specificities and functionalities such thermostability, cold activity, gene expression, solubility, enantio-selectivity, substrate specificity, and solvent tolerance [12,13]. The strategies in the directed evolution were applied to improve the thermostability of lipases such as *Yarrowia lipasea* lipase, *B. licheniformis* RSP-09 lipase and *Candida antartica* lipase B [14].

*Staphylococcus epidermidis* AT2 lipase was found to be optimally active at 25 °C and stable at cold temperatures (<30 °C) [15]. Terminal moiety mutation on wild-type AT2 lipase (rT-M386) improved its lipolytic activity by 25% at 45 °C [16]. In this study, an attempt was taken to randomly mutate the cold-adaptedrT-M386 lipase, which is produced by a mesophilic bacterium, to achieve greater thermostability. Then, the new thermostable lipase was purified and characterized to determine the impact of the mutation on the enzyme.

## 2. Results

### 2.1. Error-Prone PCR and Screening of Mutant Library

An attempt to randomly modify the structure of rT-M386 lipase was taken to achieve greater thermostability. After the transformation of the error-prone PCR mutated clones, around 1500 positive colonies were grown on tributyrin agar plate, of which 900 colonies produced bigger and more intense clearing zones on the grid plate. To isolate the colonies with thermostable enzymes, the colonies were grown on LB broth individually and the crude enzymes were tested at 50 °C. Seven mutant colonies exhibited 1.3- to 4.8-fold higher enzyme activity at 50 °C when compared to rT-M386 lipase (Table 1). The greatest improvement in activity was exhibited by mutant no. 7, which had approximately five-fold higher activity compared to the rT-M386 lipase. Therefore, the plasmid of mutant no. 7 was extracted and the presence of a lipase gene was confirmed by PCR and the sequencing result identified the location of the mutation.

### 2.2. Sequence Analysis of Mutant No. 7 to Identify Mutation Point

The nucleotide sequence of mutant no. 7 was translated to amino acid sequence via the ExPASy-Translate tool (available online: http://web.expasy.org/translate/) and aligned with the amino acid sequence of the rT-M386 lipase. A single point mutation was found at position 210 in the protein sequence, where glycine (G) had been substituted to cysteine (C). Therefore, this mutant was named as G210C. The protein sequence analysis by biology workbench (available online: http://workbench.sdsc.edu/) showed that the protein is 43 kDa, and that GST-fused protein would express as 69 kDa in pGEX-6P-1.

### 2.3. Purification of rT-M386 and G210C Lipases

**Figure 1:**
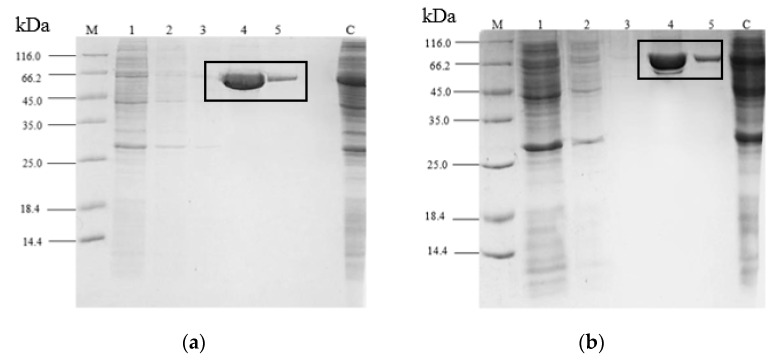

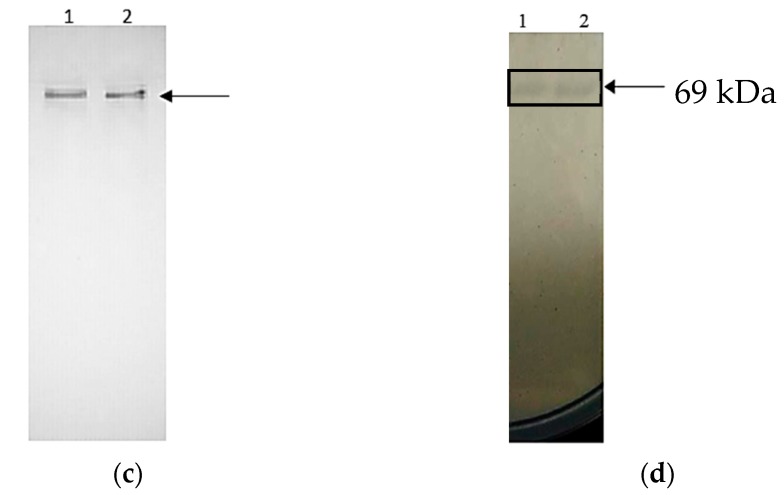
Single-step GST-purification analysis of rT-M386 and G210C. SDS-PAGE analysis of (**a**) rT-M386 and (**b**) G210C. The GST-fused lipases with the expected size of ~69 kDa are marked with a solid rectangle. Lane M represents standard protein marker. Lane 1–3: Flow-through fractions; Lane 4–5: Elution fractions; (**c**) Native-PAGE of rT-M386 and G210C; (**d**) Activity staining of rT-M386 and G210C. Lane 1: rT-M386; Lane 2: G210C. Arrows indicate the protein band.

G210C lipases were overexpressed and purified simultaneously along with rT-M386 for comparison (Table 2). The recovery for rT-M386 and G210C was 92.3% and 92.2% with the purification fold of 2.8 and 2.7, respectively. Both purified lipases migrated as a single band with a molecular weight of 69 kDa on SDS-PAGE (Figure 1).

The purification product of each lipase was further analyzed on Native PAGE. A single band showed on the gel in Figure 1c confirmed the purity and homogeneity of the protein. Zymogram was performed and the clearing zone indicates that the purified product at 69 kDa was an active lipase (Figure 1d). This further confirmed the size of the molecular weight of rT-M386 and G210C fusion lipases.

### 2.4. Characterization of Purified rT-M386 and G210C Lipases

Characterization of rT-M386 and G210C lipases proceeded immediately after a single step of purification and also without removing the GST tag.

#### 2.4.1. Effect of Temperature on Lipase Activity and Stability

**Figure 2:**
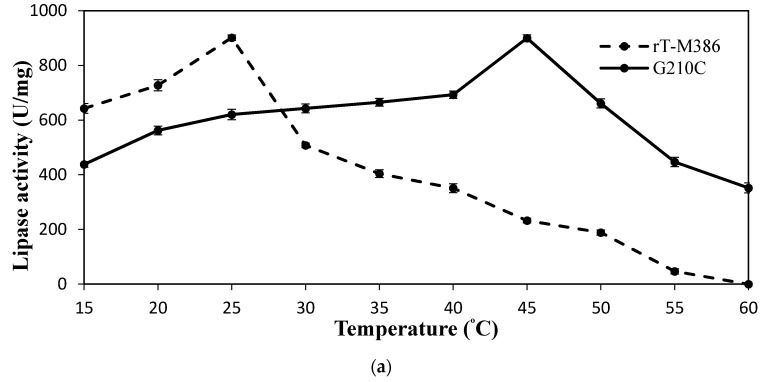

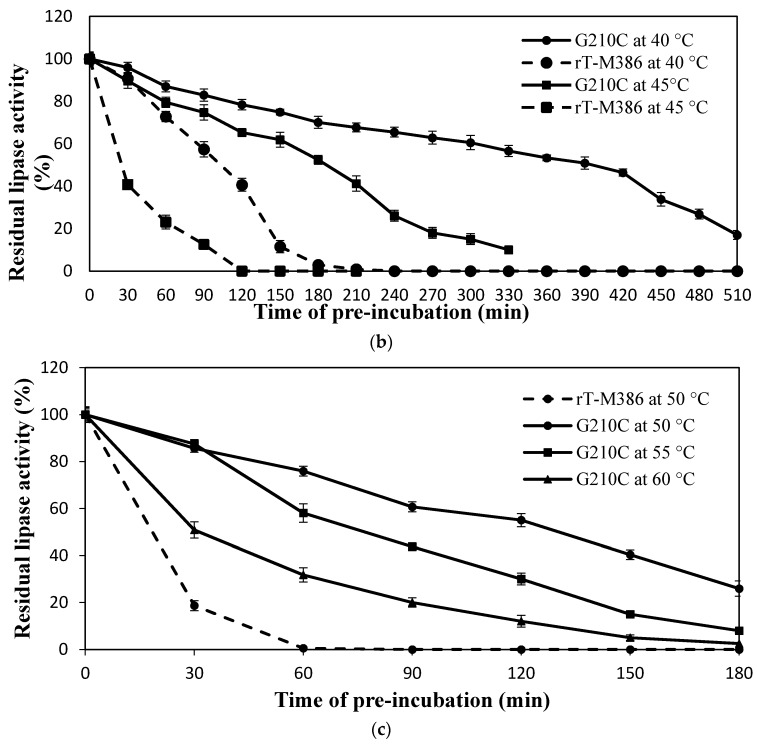
The temperature profile of purified rT-M386 and G210C lipases. (**a**) Effect of temperature on the activity of purified rT-M386 and G210C lipases. The lipases were assayed at different temperatures in the range of 15 to 60 °C; (**b**) Effect of temperature on the stability of purified rT-M386 and G210C lipases at 40 and 45 °C; (**c**) Effect of temperature on the stability of purified rT-M386 and G210C lipases at 50, 55 and 60 °C. The lipase assay for the stability test was done at the optimum temperature for each enzyme. Error bars represent the standard deviation of means (*n* = 3).

The G210C lipase was active at a temperature range of 30 to 60 °C with an optimal temperature at 45 °C with 899.7 U/mg (Figure 2a). On the other hand, the rT-M386 lipase preferred temperatures below 30 °C by exhibiting an optimum temperature at 25 °C with 901.3 U/mg and the activity dropped drastically at 45 °C (232.5 U/mg). The lipase activity of G210C at 60 °C was 351.9 U/mg while it totally diminished for the rT-M386 lipase.

#### 2.4.2. Effect of Mutation on Lipase Thermal Stability (Half-Life)

The G210C lipase displayed high stability at 40 °C as almost 70% of its original activity. Its relative activity was maintained even after 3.5 h of pre-incubation, whereas the rT-M386 lipase retained its activity only for 1 h (Figure 2b). At 45 °C, an appreciable level of stability was observed as the G210C lipase that retained 70% of its residual activity over 2 h (Figure 2b). Conversely, at 45 °C the residual activity of rT-M386 lipase was lower than 12% after 2 h. The half-life of the G210C lipase at 40 and 45 °C was 6.5 h and 3 h, respectively. Meanwhile, the half-life of the rT-M386 lipase at 40 and 45 °C was way shorter, which was less than 2 h and 20 min, respectively. Moreover, the rT-M386 lipase displayed its half-life only for 10 min at 50 °C, while the G210C lipase was stable at 50 °C with a half-life of 2.5 h (Figure 2c).

Subsequently, further treatment on the G210C lipase at 55 and 60 °C was performed up to 180 min. The rT-M386 lipase was not treated at these temperatures as it lost 82% of its original activity fairly in 30 min when subjected to 50 °C. The purified G210C lipase was stable up to 55 °C and lost only 13% of its original activity at 30 min with a half-life of nearly 1.5 h (Figure 2c). At 60 °C, the G210C lipase underwent a dramatic decrease by 49% of residual activity at 30 min.

#### 2.4.3. Effect of pH on Lipase Activity and Stability

**Figure 3:**
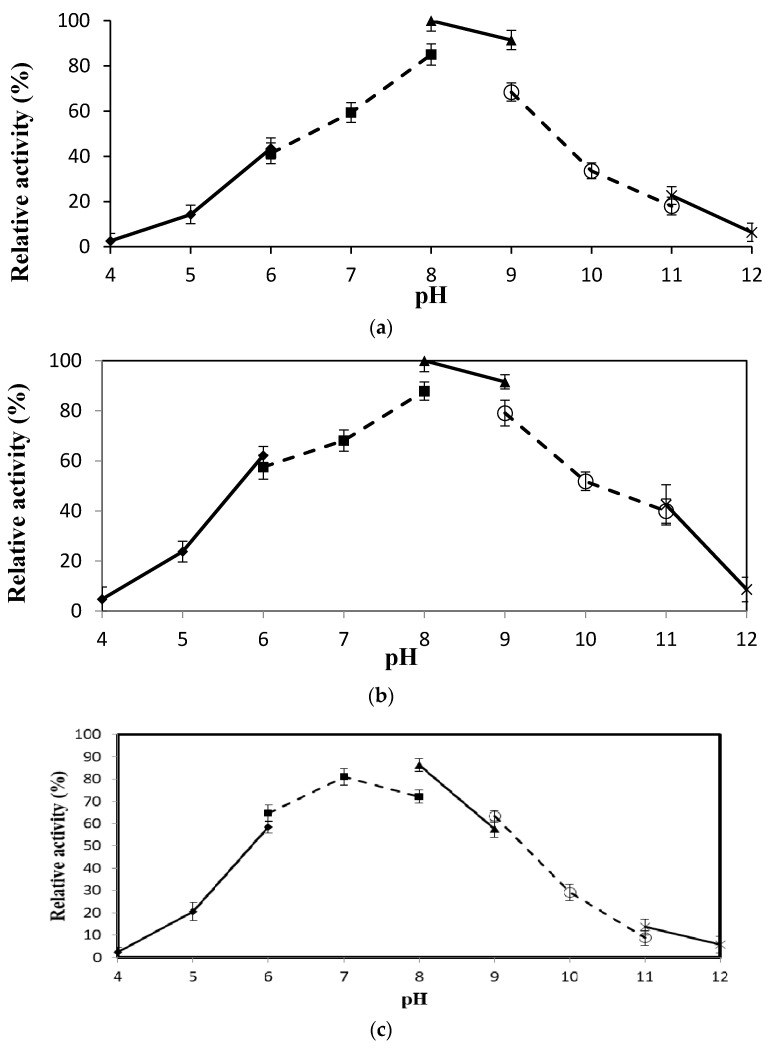

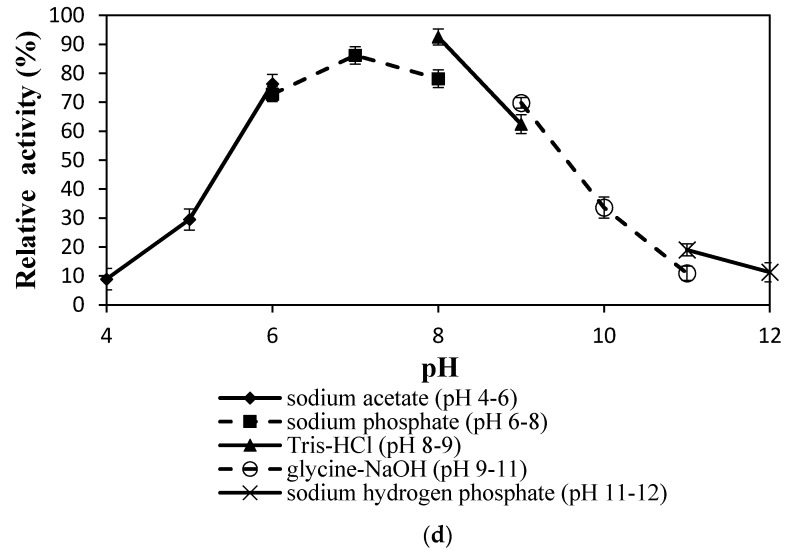
The pH profile of the lipase activity of purified rT-M386 and G210C lipases. (**a**) Effect of pH on the activity of purified rT-M386 lipase and (**b**) G210C lipase. Lipases were assayed at various pHs from pH 4 to 12 by a colorimetric method and the activities of the enzymes against different buffers, as shown as values relative to the optimum pH (Tris-HCl, pH 8.0); (**c**) Effect of pH on the stability of purified rT-M386 and (**d**) G210C lipases. Lipases were pre-incubated at various pHs from pH 4 to 12 and assay using Tris-HCl, pH 8. Error bars represent the standard deviation of means (*n* = 3).

The purified rT-M386 and G210C lipases displayed broad pH activity from pH 6 to 9 with an optimum pH of 8.0 (Figure 3a,b). The activity of the rT-M386 lipase was almost lower than 22% in sodium acetate (pH 4–5) and sodium hydrogen phosphate (pH 11–12) compared to the activity in Tris-HCl (pH 8). In addition, the G210C lipase was 42% less active in sodium acetate (pH 4–5) and sodium hydrogen phosphate (pH 11–12) compared to the activity in Tris-HCl (pH 8).

The lipases were discovered to be highly stable at pH 8. The G210C lipase and rT-M386 lipase retained 92% and 86% of their lipase activity relative to the non-incubated sample, respectively (Figure 3c,d). They were comparatively stable at pH 9 but a dramatic decrease in the stability was observed at a pH less than six and greater than nine (less than 50%).

#### 2.4.4. Effect of Organic Solvents on Lipase Activity

**Figure 4:**
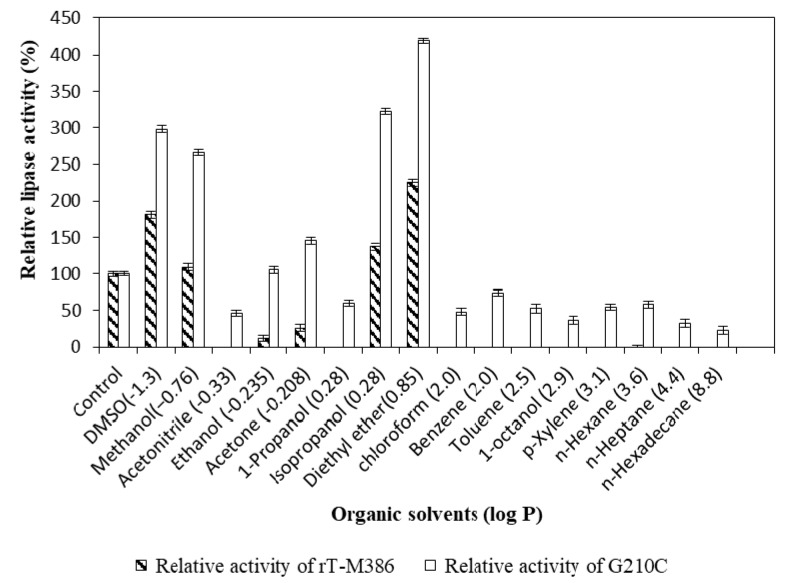
Effect of organic solvents on lipase activity. Relative activity was measured based on control sample without treatment with organic solvents. Error bars represent the standard deviation of means (*n* = 3).

The G210C lipase displayed a far better stability compared to the rT-M386 lipase. The rT-M386 lipase was only active in some of the polar solvents (log *P* < 1) and lost its activity instantaneously in non-polar solvents (log *P* > 1). The G210C lipase showed excellent activity improvement in diethyl ether, isopropanol, dimethyl sulfoxide (DMSO) and methanol (log *P* < 1) by 115–195% as compared to the rT-M386 lipase (Figure 4). For the rT-M386 lipase, a complete inhibition of activity was observed in acetonitrile and 1-propanol (log *P* < 1) and also in all the organic solvents with log *P* > 1. Meanwhile, in organic solvents with log *P* > 1, the G210C lipase retained 70% of its relative activity in benzene, almost 50% in toluene, *p*-xylene, and *n*-hexane. The G210C lipase exhibited its activity in both polar and non-polar solvents by pre-incubating at 50 °C, unlike the rT-M386 lipase.

### 2.5. Circular Dichroism (CD)Spectra Analysis of Purified rT-M386 and G210C Lipases

#### 2.5.1. Secondary Structure Analysis of Purified rT-M386 and G210C Lipases

**Figure 5:**
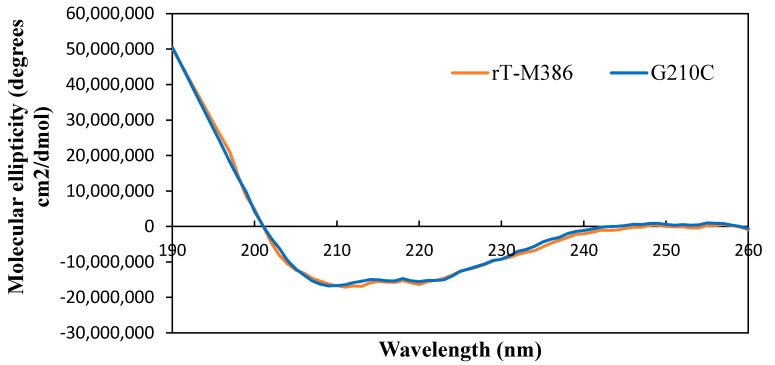
Far-UV spectra of rT-M386 and G210C lipases. Both spectra were collected at 190 to 260 nm.

The composition of the secondary structure of the rT-M386 and G210C lipases was estimated by measuring the far-UV spectra. The graph of molecular ellipticity (degrees cm^2^·dmol^−1^) was plotted against the wavelength of the spectra recorded from 190 to 260 nm (Figure 5). Principally, the trend of these far-UV spectra corresponded with the typical spectra of α-helix dominated protein structure. In addition, the constitution of α-helix was indicated when negative ellipticity was observed at 208 nm, 222 nm and also positive ellipticity at 190 nm.

There are no significant differences between the ratio of each secondary structure element in both lipases (Table 3). As an overall, all the elements of the secondary structure are evenly formed in both lipase structures.

#### 2.5.2. Thermal Denaturation of Purified rT-M386 and G210C Lipases

**Figure 6:**
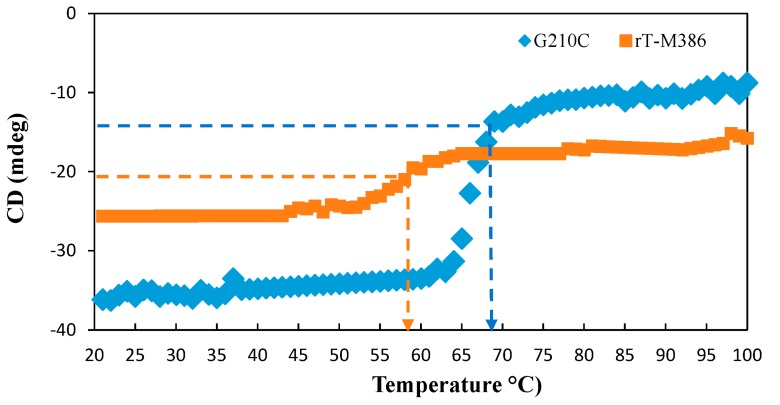
The thermal denaturation curve of rT-M386 and G210C lipases. CD spectra were measured at 222 nm between temperature ranges from 20 to 100 °C. Arrow indicates the thermal denaturation point.

The midpoint of the unfolding transition (T_m_) was measured at 222 nm, the wavelength at which the α-helix recorded a large CD signal. The T_m_ for the rT-M386 and G210C lipase was 56.38 and 66.02 °C, respectively (Figure 6). Approximately 10 °C difference of T_m_ behavior was observed between these two lipases.

### 2.6. Homology Modelling and Molecular Dynamic Simulation

#### 2.6.1. Single Point Mutation on the Structure of the G210C Lipase

**Figure 7:**
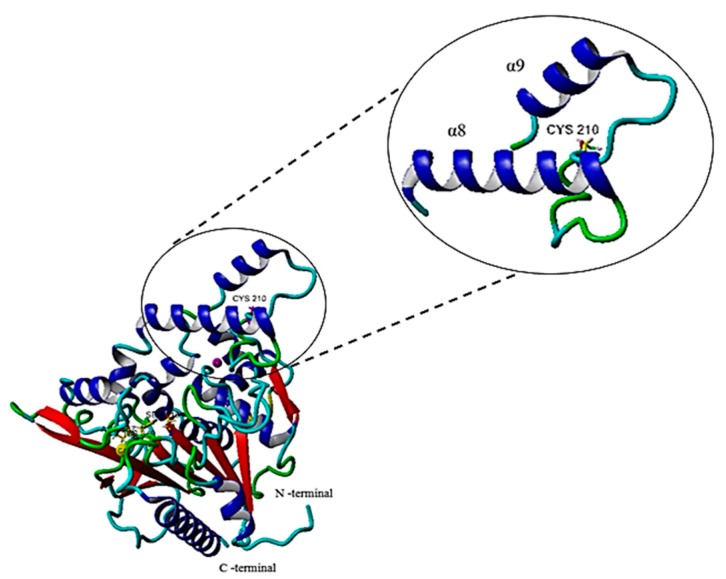
The predicted lid region of G210C lipase. Cyc 210, the mutation point, was found in the lid region between α8 and α9. The figure is generated using Yet Another Scientific Artificial Reality Application (YASARA).

The *Staphylococcus hyicus* lipase (PDB ID: 2HIH) was selected as the most suitable and reasonable template among the other homologues to predict the structure of rT-M386 and G210C lipases with a sequence identity of 51%. Both the predicted rT-M386 and G210C lipases have three catalytic residues, including Ser116, Asp307 and His349, placed at highly conserved regions residing in the loop, turn and one at a side of the β-sheet in both lipases. Most importantly, the mutation did not interrupt or make any changes to the catalytic site conformation. The G210C mutation is located in the lid region. This lid region is formed by two α-helices, α8 (lower lid) at position 181–198 linked to α9 (upper lid) at position 218–229 by the loop (Figure 7).

#### 2.6.2. Molecular Dynamic Simulation

**Figure 8:**
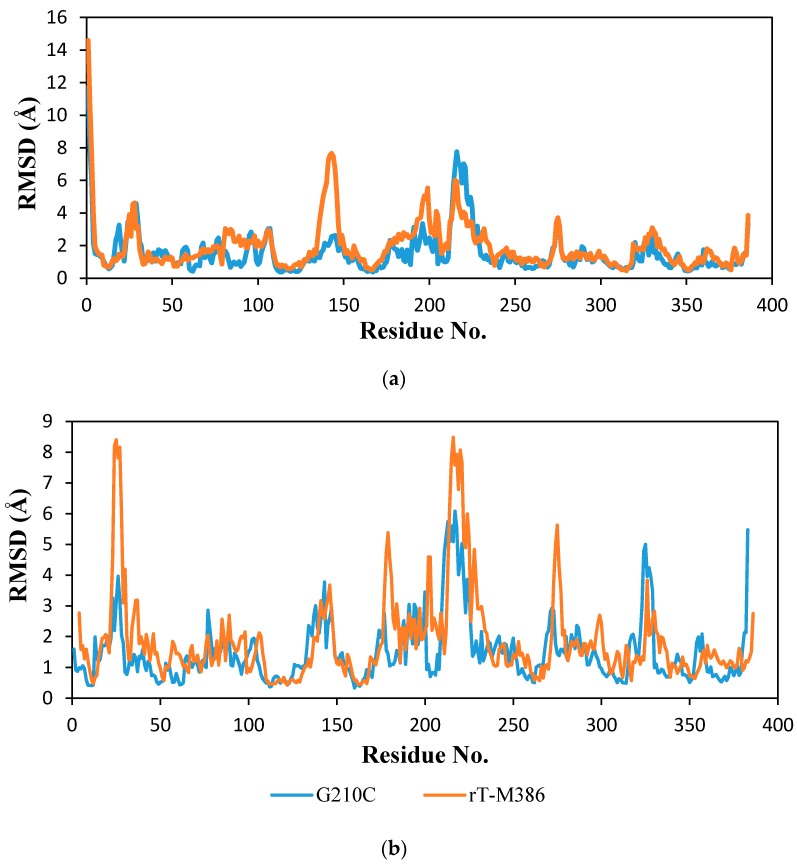
Root-mean-square deviation (RMSD) profile of the Cα backbone position for rT-M386 and G210C lipases as per residue. The RMSD values were computed at different temperatures (**a**) at 25 °C (**b**) at 50 °C. rT-M386 and G210C lipases are indicated by the red line and blue line, respectively.

The root-mean-square deviation (RMSD) of rT-M386 and G210C lipase structures was analyzed against a number of residues at 25 and 50 °C (Figure 8). The RMSD values of residue 210 at 25 °C before mutation was 2.1 Å. When glycine replaced the cysteine, the value decreased to 1.0 Å at 25 °C. Moreover, the mutation at residue 210 decreased the RMSD value further at 50 °C, where it shows only 1.4 Å. Thus, this decreasing RMSD value indicates that the lid domain (residues 181–229) of the G210C lipase has sustained a remarkable stability at elevated temperature as compared to the rT-M386 structure. Significantly, at 25 °C, several helices in the N-terminal of rT-M386 lost their stability, especially residues 82–94 and 130 to 160. Likewise, at 50 °C some regions at the N-terminal and the α/β hydrolase fold domain of rT-M386 were extremely perturbated. The N-terminal moiety of the rT-M386 lipase and the other small domains from residues 20–30 presented hypersensitivity at 50 °C as the RMSD value increased to 8.4 Å.

## 3. Discussion

The thermal stability of a lipase from *Staphylococcus epidermidis* AT2 was enhanced by conducting error-prone PCR. Construct pGEX-6p-1/M386 comprising the rt-M386 lipase gene was used as the template and proceeded to molecular cloning in order to generate a mutant library. Based on the qualitative and quantitative screening, a mutant with extremely improved thermostability was evolved. There was no cysteine residue in the protein sequence of the rT-M386 lipase. This random mutation approach introduced a cysteine, a polar residue into the sequence of the rT-M386 lipase replacing a glycine, a non-polar residue. Glycine is the smallest amino acid, with a hydrogen atom as the side chain (–H) [17]. Cysteine belongs to the sulfur amino acids and a sulfur atom appears in its side chain that is involved in the formation the sulfhydryl group (–SH), which is very reactive [18]. Cysteine composition is divided into two groups: free cysteine (–SH) and disulfide bonding half cysteine (–SS). They are dissimilar in their physio-chemical properties [19]. A disulfide bond is covalently formed between two half-cysteine residues by an oxidation reaction which contributes to the stability of a protein structure [20]. However, a free cysteine residue is capable of forming hydrogen bond with the carbonyl groups of other residues [21].

Mutation of the G210C lipase did not affect the expression level of the rT-M386 and G210C lipases. The lower lipase activity for other mutants, as compared to the G210C lipase at 50 °C, could be due to the lower expression level of those mutants. GST-tag was used fairly to carry out the purification at ease and it was completed in a single step. Dithiothreitol (DTT) or other reducing agents are optional for GST-tag chromatography if any binding or degradation problems occurred during purification. Oligomeric proteins or those with disulfide bonds in their structures may need an addition of DTT in the purification buffer. Thus, no reducing agent was used for both rT-M386 and G210C lipases to purify the enzymes. The same purification profile in SDS-PAGE and Native-PAGE was observed for both lipases. An affinity tag system allowed single-step purification with minimal effect on enzyme tertiary structure and biological activity without significantly changing the physicochemical properties [22].

Biochemical and biophysical characterization was performed to investigate the effect of mutation on the rT-M386 lipase. It was found that the optimal temperature of the G210C lipase was far higher than the rT-M386 lipase. After random mutagenesis, a 20 °C increase in optimum temperature was identified. Similarly, the optimum temperature of a thermostable variant 6B from *B. subtilis* lipase was 65 °C, which is 30 °C higher than that of wild-type lipase [23]. The activity of both lipases was inhibited at extremely acidic or alkaline pH. Generally, greater electrostatic repulsions can increase the solubility of the protein when the surrounding pH value is far beyond its isoelectric point (pI value) [24]. Therefore, both lipases were inactivated during the pH treatment far below or above its pI value. The pI value of the rT-M386 and G210C lipases were 6.45 and 6.43, respectively. The substitution of glycine to cysteine did not demonstrate any significant difference in pI value. Moreover, the G210C lipase exhibited the same numbers of charged amino acids as found in the rT-M386 lipase. Hence, both of these lipases favor Tris-HCl, pH 8 for their optimum activity; indicating that they are alkaline lipases. Generally, Staphylococcal lipases display an optimum pH of six to nine [25]. There are several reports on highly alkaline lipases in the pH range of 8.5 to 10.0, such as thermostable lipases from *Bacillus* strain A30-1 and *B. strearothermophilus* L1 [26,27]. The G210C lipase was remarkably stable in polar solvents at high temperature, in fact the activity was enhanced by 125% as compared to the control (without solvent). Similarly, the activity of thermostable lipases from *Pseudomonas* sp. S5 [28], *Arthrobacter nitroguajacolicus* Ru61 [29] and *Bacillus sphaericus* 205y [30] were inhibited by the treatment of a non-polar solvent such as hexadecane. This possibly obstructed the efficiency of the enzyme–substrate interaction due to the relatively high solvent viscosity. Conversely, significant lipase activation by the addition of polar solvents can be due to the interruption of aggregates formed between the lipase and lipids of the fermentation medium, or between the lipase molecules themselves [31].

The ratio of secondary structure in the rT-M386 is comparable with the G210C lipase, and it was estimated based on ellipticity values (Figure 5). The similarity in the ratio of secondary structure of both lipases suggested that the proportion of secondary structures is not a key factor which influences the thermal stability of the G210C lipase, but the interaction of the elements in the structure might be the factor. The sigmoidal shape of the resulting denaturation curve indicated a monophasic helix to the coil transition of a protein within the analyzed temperature range [32]. In this transition, indicating the denaturation of the protein, the conformation is either disordered or unfolded. In agreement with the experimental finding, the half-life for the G210C lipase was 30 min at 60 °C, which is close to its T_m_ value of 66.0 °C (Figure 6), while the rT-M386 lipase retained 50% of its activity for 10 min at 50 °C and lost its total activity at 60 °C.

The substitution of glycine to cysteine on the extended loop connecting α8 and α9 is an important stabilizing factor that could play a role in the protein’s ability to withstand elevated temperatures. The comparative analysis of the average RMSD values at 50 °C conferred a higher value (2.6 Å) on the rT-M386 than the G210C structure (1.9 Å), suggesting that the mutant had the greater stability due to conformational rigidity. In addition, increased rigidity of enzymes is required to achieve high stability [33].

In conclusion, this directed evolution technique was proven to be extremely efficient for the engineering of the lipase in a single round of error-prone PCR to improve catalytic performance in high temperatures and harsh conditions. The introduced residue substitution in the lid region most probably re-stabilized the loop to provide rigidity to the structure of the G210C lipase, which was also supported by the experimental result. The G210C lipase has manifested its property as a thermostable and organic-solvent-tolerant lipase that makes it suitable for industrial applications.

## 4. Materials and Methods

### 4.1. Bacterial Strains, Plasmid, and Media

*Staphylococcus epidermidis* AT2 was isolated from the contaminated soil at a car service area in Port Dickson, Malaysia [34]. The C-terminal truncated gene was cloned into pGEX-6p-1 expression vector and transformed into *E. coli* BL21 (DE3) (Novagen, Darmstadt, Germany) [16]. The bacterial culture was cultivated in Luria-Bertani (LB) medium (Difco, Sparks Glencoe, MD, USA), and supplemented with 50 μg/mL of ampicillin (Sigma, St. Louis, MO, USA). Restriction enzymes and plasmids were purchased from Thermo Scientific. All the chemicals used were of analytical grade.

### 4.2. Error-Prone PCR and Generation of Mutant Library

The mutant library was generated via error-prone PCR using the GeneMorph^®^ II Random Mutagenesis kit (Stratagene, La Jolla, CA, USA) according to the manufacturer’s instructions. The plasmid of rT-M386 was used as a template with forward (5′-CGGAATTCGCAGCAGCAATGGCGCAAGCTCAATATAAAAAT-3′) and reverse primers (5′-CCGCTCGAGTCACTAACCATCTAGCTCTTCGACTTTCAA-3′). The amplified PCR products and pGEX-6P-1 vector were double digested, ligated and then transformed into *E. coli* strain BL21 (DE3). The transformed cells were plated on a tributyrin-ampicillin agar plate.

### 4.3. Screening and Selection of Mutants

The resulting mutant library was screened for thermostable lipase mutants by a three-step screening method. First, tributyrin-ampicillin plates were used to grow all the cells harboring mutant clones. The plates were incubated for 12 h at 37 °C, because this is the optimum temperature for the growth of *E. coli* cells. This preliminary screening of mutant clones was done by observing the formation of a clearing zone around the colony on tributyrin-ampicillin agar plate. In the second step, the positive mutant colonies were picked using sterile toothpicks, placed on the grid plates and incubated at 37 °C for 12 h. Then, the colonies with bigger and more intense clearing zones were screened and the enzymes were expressed in a small scale (Section 2.4). As for the third screening step, the crude lipase extract of each clone was subjected to lipase activity assay at 50 °C in order to identify the mutants with a higher index of thermostability compared to the rT-M386 enzyme (Section 2.5). The protein concentration of all mutants was measured by Bradford assay [35] and standardized prior to lipase assay.

### 4.4. Expression of Soluble Proteins of Mutants

Selected mutants were grown in 50 mL sterilized LB broth with 50 μg/mL ampicillin at 37 °C in the incubator shaker (200 rpm) for 2–3 h. Then, the bacteria were induced with 0.25 mM IPTG at OD_600_ = 0.6 and incubated at 25 °C for 16 h. The cells were centrifuged at 10,000× *g* for 10 min at 4 °C. The supernatant was discharged and the pellet was resuspended in 10 mL 50 mM Tris buffer (pH 8) prior to sonication. Sonication was carried out for 4 min with 30 s interval on ice by using Branson Sonifier 250 (output two, duty cycle: 30) (Branson, Danbury, CT, USA). The cells were then centrifuged at 10,000× *g* for 30 min. The supernatant is the soluble protein which was used for the colorimetric assay.

### 4.5. Lipase Activity Assay

Lipase activity was determined by the modified method of Ertuğrul et al. [36] using p-nitrophenyl palmitate (pNPP) (Sigma) as substrate. The substrate solution was prepared by freshly mixing 25 μL of 25 mM pNPP with 950 μL of 50 mM Tris-HCl buffer pH 8 containing 0.1% (*w*/*v*) of gum arabic. The mixture of 975 μL of substrate solution and 25 μL of enzyme was incubated at 25 °C for 10 min. The enzyme reaction was terminated by adding 250 μL of 3 M NaOH. Then, lipolytic activity was determined by measuring the absorbance at 410 nm. One unit (U) of enzyme activity was defined as 1 μmol of *p*-nitrophenol released per minute under the assay conditions.

### 4.6. Sequencing and Sequence Analysis

Mutants which are selected based on colorimetric assay were proceeded with sequencing to identify the location of the mutation. Recombinant plasmids of the selected mutants were sequenced by MyTACG Bioscience, Malaysia. Subsequently, the obtained sequence of mutants was analyzed and aligned with the sequence of rT-M386 lipase by using Biology Workbench 3.2 (available online: http://workbench.sdsc.edu/).

### 4.7. Purification of rT-M386 and G210C Lipases

The selected mutant (G210C) and rT-M386 lipases were purified individually by a single step of affinity chromatography using Glutathione Sepharose resin (GE Healthcare, Aurora, OH, USA). The soluble crude enzyme was loaded into the column and flowed at 1 mL/min.The column was washed with binding buffer (50 mM phosphate buffer, pH 7.4) until no protein was detected through A_280_ nm readings. The bounded protein was eluted isocratically using elution buffer composed of 50 mM Tris HCl, pH 8.0 and 10 mM reduced glutathione with a flow rate of 2 mL/min. The fractions (5 mL) that exhibited lipase activity were collected. The target protein was dialyzed against 50 mM Tris-HCl buffer, pH 8, prior to characterization.

The eluted peak was subjected to SDS-PAGE (12%) under the conditions developed by Laemmli [37]. The sample was mixed with the 4× sample buffer in 3:1 ratio. The electrophoresis was run on BioRad’s Mini Protean II, BioRad USA at 200 V for 45 min with 30 mA constant current. The protein bands were visualized by a staining solution composed of 10% (*v*/*v*) acetic acid, 45% (*v*/*v*) methanol and 1% (*w*/*v*) Coomassie Brilliant Blue R250 for 10–15 min and de-stained by 10% (*v*/*v*) methanol and 10% (*w*/*v*) acetic acid solution. The band corresponding to the lipase was determined by zymogram of the unstained gel. The purified protein (without boiling during sample preparation) was electrophoresed on SDS-PAGE. The gel was immersed in 20% isopropanol (30 min) to remove SDS from the gel. Then, the gel was rinsed with distilled water two times before placing it on a tributyrin agar plate and incubated at 25 °C for 1–2 h. A clearing zone was formed on the tributyrin agar plate indicating the band of a target protein with lipase activity. To confirm the homogeneity of the target protein, the purified protein was electrophoresed on Native-PAGE and the protein band on the gel was visualized using Coomassie Brilliant Blue R250.

### 4.8. Biochemical Characterization

The lipase characterization was performed using the purified enzymes with a protein concentration of 0.5 mg/mL. Each experiment was done in triplicate and values are presented as mean ± SD, unless otherwise stated.

#### 4.8.1. Effect of Temperature on Lipase Activity and Stability

The effect of temperature on rT-M386 and G210C lipase activity was measured using p-nitrophenyl palmitate (pNPP) at different temperatures ranging from 15 to 60 °C at 5 °C intervals according to the method described in Section 2.5. The thermostability of the rT-M386 and G210C lipases was determined by pre-incubating the rT-M386 lipase at 40, 45 and 50 °C for 8.5, 3.5 and 3 h, respectively, and G210C lipase at 40, 45 and 50 °C for 8.5, 5.5 and 3 h, respectively. Additionally, the thermostability of G210C lipase was tested at 55 and 60 °C by pre-incubating for 3 h. All pre-incubated samples were rapidly cooled and assayed at the optimum temperature according to the modified method of Ertuğrul et al. [36]. The residual activity of each sample was measured as a percentage compared to the activity of sample assayed at the optimum temperature without incubation, considered as 100%.

#### 4.8.2. Effect of pH on Lipase Activity and Stability

To determine the optimal pH of rT-M386 and G210C, the lipases were assayed at optimum temperature (25 and 45 °C for rT-M386 and G210C, respectively) with different pHs ranging from pH four to 12. The buffer systems involved 50 mM acetate buffer for pH 4–6, potassium phosphate buffer for pH 6–8, Tris-Cl buffer for pH 8–9, glycine-NaOH for pH 9–11, and Na_2_HPO_3_/NaOH buffer for pH 11–12. A pH stability test was performed by pre-incubating the rT-M386 and G210C lipases for 30 min with different pH values at 25 and 45 °C for rT-M386 and G210C, respectively. The samples were subjected to lipase assay calorimetrically after pH treatment. The relative activity of each sample was measured as a percentage compared to the activity of the sample assayed without incubation.

#### 4.8.3. Effect of Organic Solvents on Lipase Activity

Purified rT-M386 and G210C lipases were pre-incubated at 50 °C for 30 min with different organic solvents (50% *v*/*v*); DMSO (−1.3), methanol(−0.76), acetonitrile (−0.33), ethanol (−0.235), acetone (−0.208), 1-propanol (0.28), isopropanol (0.28), diethyl ether(0.85), chloroform (2.0), benzene (2.0), toluene (2.5), 1-octanol (2.9), *p*-xylene (3.1), *n*-hexane (3.6), *n*-heptane (4.4), and n-hexadecane (8.8).Then, the lipase assay was conducted at 25 and 45 °C for rT-M386 and G210C, respectively. The stability was determined relatively to the control without the organic solvent, considered as 100%.

### 4.9. CD Spectra Analysis of Purified rT-M386 and G210C Lipases

Biophysical characterization and thermal denaturation of the lipases were analysed through circular dichroism (CD) spectroscopy. The purified rT-M386 and G210C were prepared in sodium phosphate buffer (10 mM, pH 7.0) and analysed with spectropolarimeter J-815 (Jasco, Tokyo, Japan). The machine was warmed-up with the periods of 50 to 95 °C for 30 min. Each step was one degree per min and a wavelength scan of 190 to 260 nm was taken into consideration.

#### 4.9.1. Secondary Structure Analysis of Purified rT-M386 and G210C Lipases

Secondary structure analysis was carried out to predict the percentages of each secondary structural element in the structure of rT-M386 and G210C lipases. The concentration of lipases was 0.03 mg/mL. The far-UV measurement was collected from 190 to 260 nm using a 0.1 cm path-length quartz cuvette.

#### 4.9.2. Thermal Denaturation of Purified rT-M386 and G210C Lipases

The variable temperature measurement ranging from 20 to 100 °C of lipases was performed by employing 0.1 cm path-length quartz cuvette. The wavelength was set to 220 nm. The concentration of each lipase was 0.1 mg/mL. The top of the cuvette was completely closed using a cap to minimize the evaporation. Data pitch, bandwidth, scanning speed, and accumulation were set to be 1.0, 1.0, 100 nm per min, and three times, respectively.

### 4.10. Homology Modelling and Molecular Dynamic Simulation

The rT-M386 and G210C lipase sequences were modeled using a crystal structure of *S. hyicus* lipase (PDB ID: 2HIH) with a sequence identity of 51% as the template using Yet Another Scientific Artificial Reality Application (YASARA).

Simulations were applied to rT-M386 and G210C lipase structures in the AMBER03 force field YASARA. The periodic box was filled with explicit water molecules to a density of 0.98320 g/L. The whole system was neutralized by adding counter ions and all ionizable protein groups were protonated according to their tabulated pKa values at pH 7 of the medium. Water molecules were relaxed by a simulated annealing procedure. Minimization was run until the maximum atom speed dropped below 2200 m/s. Then the system was heated from 0 to 298 K (25 °C) and 323 K (50 °C).

In total, 20,000 ps molecular dynamics (MD) equilibrated simulation was conducted at 298 and 323 K in constant pressure. MD simulations were carried out at long simulation times to ensure that the protein atoms reached an equilibrated state. The cutoff was 7.86 Å for van der Waals interactions. The electrostatic interactions were calculated without cutoff by particle mesh Ewald algorithm. During the simulations, three-dimensional (3D) coordinate snapshots were collected every 0.25 ps interval. Kinetic energy was initialized on each replica assigning random velocity vectors to all atoms (using Maxwell_Boltzman distribution). YASARA analysis toolkit utilities were used to analyze MD trajectories and a table was created with average root mean standard deviation (RMSD). For plotting graphs, Microsoft Office Excel 2016 was used (Microsoft Corporation, Tulsa, OK, USA).

## Figures and Tables

**Table 1:** Specific activity of rT-M386 and mutant clones at 50 °C.

Lipases	Specific Activity (U/mg) ± SD	Fold
rT-M386	11.82 ± 2.47	1.0
Mutant no.1	17.82 ± 2.38	1.5
Mutant no.2	15.65 ± 2.01	1.3
Mutant no.3	31.98 ± 2.08	2.7
Mutant no.4	35.15 ± 2.04	2.9
Mutant no.5	27.82 ± 2.11	2.3
Mutant no.6	22.48 ± 2.30	1.9
Mutant no.7	56.65 ± 2.88	4.8

Note: Values are means of three replicates ± SD.

**Table 2:** Purification table of (a) rT-M386 (b) G210C.

(**a**)					
**Step**	**Total Activity (U)**	**Total Protein (mg)**	**Specific Activity (U/mg)**	**Yield (%)**	**Purification Fold**
Crude Extract	8258.4	23.77	347.46	100	1
GST-Affinity	7618.4	8.40	906.39	92.17	2.63
(**b**)					
**Step**	**Total Activity (U)**	**Total Protein (mg)**	**Specific Activity (U/mg)**	**Yield (%)**	**Purification Fold**
Crude Extract	8178.40	32.89	248.62	100	1
GST-Affinity	7538.40	12.21	617.64	92.25	2.78

**Table 3:** Secondary structure composition of rT-M386 and G210C lipases.

Ratio (%)	rT-M386	G210C
Helix	18.4	17.3
Beta	28.3	26.2
Turn	20.9	21.1
Random	32.4	35.4
Total	100	100
